# Case Series of *Mycobacterium Abscessus* Infections Associated with a Trigger Point Injection and Epidural Block at a Rural Clinic

**DOI:** 10.4178/epih/e2012001

**Published:** 2012-01-25

**Authors:** Jun Young Song, Jung Bin Son, Min Ki Lee, Jin Gwack, Kil Soo Lee, Ji Young Park

**Affiliations:** 1 Epidemic Intelligence Service, Division of Public Health Administration, Gyeongnam Provincial Office, Changwon, Korea.; 2 Department of Internal Medicine, Pusan National University School of Medicine and Medical Research Institute, Busan, Korea.; 3 Division of Epidemic Intelligence Service, Korea Centers for Disease Control and Prevention, Osong, Korea.; 4 Division of Bacterial Respiratory Infections, Center for Infectious Diseases, National Institute of Health, Korea Centers for Disease Control and Prevention, Seoul, Korea.; 5 Department of Internal Medicine, Kosin University College of Medicine, Busan, Korea.

**Keywords:** Epidural block, *Mycobacterium abscessus*, Outbreak, Trigger point injection

## Abstract

**OBJECTIVES:**

The aim of this report is to investigate *Mycobacterium abscessus* infections at a rural clinic and carry out a surveillance program to determine the extent and source of these infections.

**METHODS:**

The authors conducted an active surveillance investigation of 36 patients who had visited the clinic since 1 July 2008. Clinical specimens were collected from the patients and an envirnmental investigation. Pulsed-field gel elctrophoresis (PFGE) was performed for comparing with *M. abscessus* isolates from the patients.

**RESULTS:**

Six specimens were obtained from the 6 patients respectively and 22 environmental samples were obtained. *M. abscessus* was isolated from the wounds of two patients, and various nosocomial pathogens, but not *M. abscessus*, were isolated from the surrounding environment. Two strains of *M. abscessus* from patients were identical as a result of PFGE.

**CONCLUSION:**

Infection control education including proper hand hygiene should be emphasized for physicians performing invasive procedures. There also needs to be more attention for invasive procedures management, including trigger point injection and epidural block in rural clinics.

## INTRODUCTION

Nontuberculous mycobacteria (NTM) are found in the natural environment throughout the world and include at least 65 different species [1]. NTM exist widely in soil and water and are known to colonize and cause disease in a broad range of animal species. There is sufficient evidence that nosocomial transmission of these organisms is increasing [2,3]. *Mycobacterium abscessus* is an acid-fast bacillus (AFB) classified as a pathogenic "rapid growing" strain of NTM [2,4]. *M. abscessus* was first reported by Moore and Frerichs in 1953 [5]. Although *M. abscessus* is an uncommon causative pathogen of human disease, it can cause skin and soft tissue infections (SSTI) on damaged skin lesions following inoculation, minor trauma or surgery [5,6].

Trigger point injection (TPI) is widely used for treatment by some physicians to reduce myofascial pain and headache that is unresponsive to medical treatments in Korea [7,8]. Trigger points may irritate the nerves around them and cause referred pain, or pain that is felt in another part of the body. Lidocaine or steroid mixed normal saline injection with a 24 or 26 gauge needle to the trigger point helps to reduce pain. Many surveys regarding the safety of this procedure have been conducted but the effectiveness of TPI is controversial for a decade [9].

There have been few reports of *M. abscessus* outbreak associated with TPI [10-12] and/or epidural block (EB) procedures over the past years. The aim of this study was to investigate *M. abscessus* infections at a rural clinic and carry out a surveillance program to determine the extent and source of these infections.

## MATERIALS AND METHODS

### Outbreak recognition

On 2 March 2009, a patient (index case) who was referred from a local clinic in Ha-dong-gun province, was diagnosed with an NTM infection in hospital A and was reported to the Korea Centers for Disease Control and Prevention (KCDC). He had multiple nodules and abscesses following EB procedure at a rural clinic in mid-October 2008. The lesions were initially treated with antibiotics, abscess incision and drainage, without improvement.

On 9 March 2009, the team from KCDC, the public health center and the provincial government performed an epidemiological investigation at the clinic in Ha-dong-gun province and interviewed the physician who performed the TPI and/or EB procedures. In the field, our team was notified that similar patients had been treated in another hospital B. These events prompted the initiation of an epidemiological study to investigate the outbreak.

From 1 July 2008 to 28 February 2009, 496 patients had visited the clinic in Ha-dong-gun province. We attempted to contact all 496 patients; 428 by telephone and 68 by mail. Our team made contact with 426 patients and found 53 patients had symptoms of a skin and soft tissue infection. Of these 53 patients, 36 (68.0%) agreed to participate in the present study. They were notified of a suspicious outbreak of *M. abscessus* infection following TPI and/or EB procedures at the clinic and the danger of the infection and interviewed by telephone with a standardized questionnaire.

### Case identification

Thirty-six patients agreed to participate in the present study were classified into two categories. A confirmed case was defined as a SSTI diagnosed after 1 July 2008 and due to *M. abscessus* infection following a TPI and/or an EB at the clinic. A probable case was defined as a SSTI, diagnosed after 1 July 2008, following a TBI and/or an EB in the clinic that did not respond to standard antibacterial therapy.

Active surveillance of patients by medical record survey and telephone who had visited the clinic since 1 July 2008 was conducted to define the extent of the outbreak. Board-certified physicians interviewed potential case patients using a standardized questionnaire to obtain data on demographic characteristics, pertinent details of the TPI and/or EB, additional procedures afterwards, the date of symptom onset, symptoms and treatment. The study protocol was approved by the Institutional Review Board at KCDC.

### Environmental investigation

On 9 March 2009, 22 environmental specimens were collected from the clinic for mycobacterial culture. Specimens were taken from all devices and materials within the clinic that could have come into contact with the patient's skin before, during and after the TPI and/or EB procedure session, including pillows, beds, blankets, humidifier, air conditioner, front desk, telephone and refrigerator. Water samples were taken from the sink, water bucket, bathroom, and faucets. The samples, including disposable syringes, water for injection, drugs for injection may not accurately represent the environmental conditions at the time of infection.

### Laboratory testing

Clinical specimens were obtained from 6 patients. In 2 patients, pus and exudates samples were collected from the infected wounds of the thigh and waist by sterile swabs respectively. In 2 patients, pus sample were collected from the infected wound of the waist and neck by needle-aspiration respectively. In the remaining 2, in addition, excision skin biopsies were done from the thigh and chest.

Specimens were prepared as described previously [13,14]. Species identification for these isolates was done by means of comparative sequence analyses of portions of the 16S rRNA gene, *hsp65* and *rpoB*, which were amplified by polymerase chain reaction (PCR) [15,16].

Randomly amplified polymorphic DNA (RAPD) PCR, using three primers, INS-2, IS986-FP, URP-6, as described by Zhang et al. [17] with modification was performed on patient and environmental samples.

Samples then underwent pulsed-field gel electrophoresis (PFGE) analysis. The large restriction fragments were divided and digested with Asel, as previously described [18].

Isolates were tested for susceptibility against amikacin, cefoxitin, ciprofloxacin, clarithromycin, doxycycline, imipenem, linezolid, tobramycin and moxifloxacin by means of a broth microdilution method at the National Institute of Health [19].

### TPI and EB procedure description

Twenty-one patients (58.3%) were disinfected with 75% ethanol swabs before a TPI and 17 patients (47.2%) were disinfected again after the TPI with 75% ethanol swabs. Before an EB, the skin was swabbed with povidone iodine and was not re-swabbed after an EB. After the skin was swabbed with 75% ethanol, a TPI was performed using disposable needles without time to dry. Physician applied the antiseptic using concentric circles from the center toward the outside. He used a glove only his left hand without performing hand hygiene. Epidural needle and spinal set were recycled after sterilization by autoclave. Drugs used for the TPIs were lidocaine, normal saline and dexamethasone. Lidocaine and normal saline were used for the EBs. High-frequency therapy was administered after a TPI, but not after an EB. Hot pack s were not used with any patients

## RESULTS

### Case patients

The clinic was opened from 7 May 2005 to 1 March 2009. Most patients visited the clinic with neuromuscular pains. Of the 36 patients interviewed, Thirteen (36%) were male and 23 (64%) were female. The median age was 56 years (range 34 to 81 years). Underlying diseases were diabetes (n=4, 11.1%), hypertension (n=4, 11.1%), cerebral infarction (n=1, 2.8%) and malignant disease (n=1, 2.8%).

Of the 36 patients, 29 (80.6%) underwent combined TPI and EB procedures at the clinic. Five (13.9%) underwent TPI only, while two (5.6%) underwent an unknown procedure. *M. abscessus* was isolated from the wound swab and needle aspiration of two patients respectively (5.6%). The average number of needle passages per day was as follows: 1 to 9 (27 patients, 75.0%) and 10 and over (9 patients, 25.0%). Five patients (13.9%) were treated with high-frequency therapy after their TPI and EB procedures.

**Figure 1:**
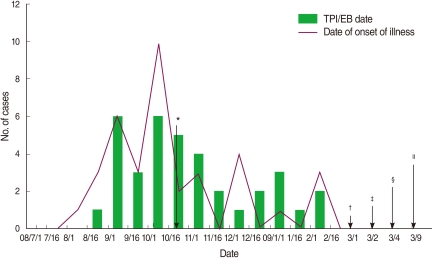
Epidemic curve of *Mycobacterium abscessus* infections following trigger point injection and/or epidural block. The mean incubation time was 5.5 days (range 1 to 15 days). TPI, trigger point injection, EB, epidural block. ^*^Index case presented with a skin lesion about two weeks after having a TPI and an EB in mid-October 2008; ^†^The local clinic was closed; ^‡^First patient was reported; ^§^A formal epidemiological study was initiated; ^∥^The physician at the clinic in Hadong-gun was interviewed. 22 environmental specimens were collected from the clinic.

Thirty-six patients who underwent TPIs and/or EBs between 1 July 2008 and 28 February 2009 subsequently had skin lesions or abscesses at the injection sites. The outbreak ceased at the end of February 2009 due to the closure of the clinic (Figure 1).

### Clinical characteristics

Ten patients (27.8%) had systemic symptoms, such as a fever or chills, and two patients (5.6%) had lymphadenopathy around their skin lesions. Out of 36 patients with papular skin lesions, 23 complained of a burning sensation around the skin lesion and 35 had erythematous papular skin lesions. Thirty-six patients (100%) had skin lesions corresponding to the injection site. The lesion sites were located as follows: thigh (n=20, 55.6%), waist (n=12, 33.3%), knee (n=12, 33.3%), shoulder (n=9, 25.0%), buttocks (n=7, 19.4%), hand (n=5, 13.9%), back (n=2, 5.6%), wrist (n=2, 5.6%) and neck (n=1, 2.8%). In addition, the mean number of lesions was 2.9 (range 0 to 30). The mean incubation time was 5.5 days (range 1 to 15 days). Clinical characteristics are summarized in Table 1.

### Environmental investigation

The environmental samples were obtained on 9 March 2009. The samples may not have accurately represented the environmental conditions at the time of the outbreak because the clinic was closed due to private problems before the investigation. M. abscessus was not isolated from the 22 environmental samples taken from the clinic. However, most cultures were contaminated by various nosocomial pathogens, including several NTM species such as *Mycobacterium septicum*, *M. avium* and *M. mageritense*.

### Microbiologic examination

**Figure 2:**
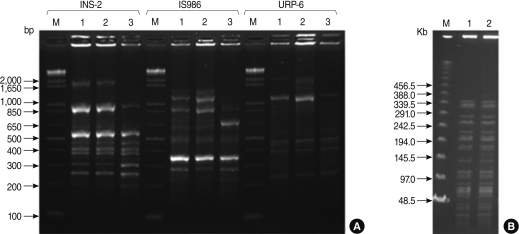
Molecular strain typing of isolates by randomly amplified polymorphic DNA PCR (RAPD-PCR) (A) and pulsed-field gel electrophoresis (PFGE) (B). (A) DNA fragments amplified by RAPD-PCR using three primers, INS-2, IS986-FP, URP-6. Lane M, 1Kb marker (Solgent); Lane 1, patient isolate in hospital B; Lanes 2, patient isolate in hospital C; Lanes 3, *M. abscessus* ATCC19977 (Type strain of *Mycobacterium abscessus*) control. (B) PFGE patterns of genomic DNA digested with Asel. Lane M, Lambda ladder PFG marker (NEB); Lane 1, patient isolate in hospital B; Lane 2, patient isolate in hospital C. Both of the isolates from the two patients had identical patterns. PCR, polymerase chain reaction.

Six specimens were obtained from the 6 patients respectively in four different hospitals (A, B, C and D). Mycobacteria were isolated from the wounds of two patients in two hospitals respectively (B and C). For these isolates, nucleotide sequences of the portions of the 16S rRNA, *hsp65* and *rpoB* were determined and analyzed to identify the isolates as *M. abscessus* (Data not shown). Two isolates were identified as *M. abscessus*, with the same sequence analysis. Two isolates also were determined by RAPD-PCR and the isolates were identified as *M. abscessus* (Figure 2A).

One was a patient's specimen which was collected from the wound swab in hospital B on 9 March 2009. The other specimen was collected from the wound needle aspiration in another hospital C. *M. abscessus* isolates from the two patients' skin wounds were compared using RAPD-PCR and PFGE analysis. RAPD-PCR and PFGE analysis resulted in an identical pattern among two *M. abscessus* strains (Figure 2B), implying that a single strain of *M. abscessus* was involved in the outbreak. The other four specimens were not identified a *M. abscessus*.

Both of the isolates showed identical antimicrobial susceptibility profiles. They were sensitive to amikacin and clarithromycin, but resistant to doxycycline and ciprofloxacin.

### Treatment and outcomes

Thirty-three (91.7%) of 36 patients were admitted to the different hospitals. Among them, 13 patients (39.4%) had undergone incision and drainage and two patients (6.1%) underwent aspiration of the lesions. The other 18 patients (54.5%) were given only antibiotic therapy. Ten of 18 patients were treated administration of oral clarithromycin (500 mg, twice daily) for 4-6 months and the other patients also received either amikacin or a combination of clarithromycin and ciprofloxacin. All admitted patients recovered fully during the investigation and no deaths occurred.

## DISCUSSION

Outbreaks of mycobacteria have been reported following injections of histamines, lidocaine, saline solution, vaccines, disinfectant solution and adrenal cortex extracts [10,20,21]. Injection safety is an enormous global health challenge both in the developing world, where safe injection practices are often lacking, and in developed countries, where new technologies and the transition of clinical practice to less regulated outpatient settings is occurring [22-24]. With the increase of TPI and/or EB procedures, adverse events also have been reported, such as needle pain, tiredness, bleeding, headache, faintness, and rarely, pneumothorax [25,26].

Thirty-six patients who underwent TPIs and/or EBs at a same rural clinic between 1 July 2008 and 28 February 2009 subsequently had skin lesions or abscesses at the injection sites The symptoms seen in our cases is similar to that reported in previous outbreaks of *M. abscessus* infection following acupuncture in Korea [14,27]. Erythematous nodule or mass, a finding reportedly suggestive of *M. abscessus* infection following acupuncture [14], were seen in almost all of our patients. In our cases, *M. abscessus* was isolated from two patients only in two hospitals (B and C). Two strains of *M. abscessus* from patients were identical using PFGE analysis. Moreover, the 2 isolates were compared with use of RAPD-PCR with reference to type strain of *M. abscessus* (*M. abscessus* ATCC 19977) because almost 50% of strains cannot be assessed by PFGE [17]. Two methods resulted in an identical pattern among the 2 isolates.

There were several limitations in identifying the exact contamination source. Because the clinic was closed due to private problems before the completion of the investigation, the environmental sources for the rapidly growing mycobacteria and their mechanism of distribution could not be elucidated.

However, there are several possible sources of the outbreak. First, inadequate sterilization of the injection sites could be the source of the infection. Because 75% ethanol and povidone iodine have a wide-spectrum germicidal effect, they are commonly used as a disinfectant for procedures. When improperly used, however, the mixture is not as effective as a disinfectant. A considerable amount of time is necessary for the prevention of mycobacterial infections after 75% ethanol or povidone iodine is applied [28]. Second, insufficient equipment sterilization and the clinic's general environment could be the source of the infection. The equipment, including epidural needle, spinal set were sterilized with an autoclave by a nurse who works in a clinic without precise protocol of sterilization. A third possibility is improper preparation of the injection drugs and septic techniques. Drugs used for the TPIs were lidocaine, normal saline and dexamethasone, but dexamethasone was not used for the EBs. Since the middle of October 2008, the physician performed the TPIs drugs, including lidocaine and normal saline, except dexamethasone. After finishing the treatment of the last patient each day, the physician prepared the following day's injection drugs and placed them in a refrigerator for up to two days. When the outbreak began, the physician had been preparing the injection drugs and placing them in a refrigerator for up to two days. The drugs were not sterilized. The samples, including disposable syringes, water for injection, drugs for injection may not accurately represent the environmental conditions at the time of infection because the clinic was closed due to private problems before the completion of the investigation. Although contaminated drugs were not determined as the source of the outbreak, the long-term storage of the injection drugs could be implicated. Additionally, whenever the physician performed TPI and/or EB, he used a glove only his left hand without performing hand hygiene; creating yet another potential source of infection. Moreover, the physician responsible for the injections used multiple-dose bottles (100 mL) of normal saline in order to dilute lidocaine and dexamethasone. Yuan et al. [11] reported cases of post-injection abscesses from extrinsic contamination of multiple-dose bottles of normal saline.

Another limitation was the difficulty in conducting the epidemiological study. Most of the 36 patients with symptoms had recovered before the study, so we obtained only six specimens in four hospitals (A, B, C and D). Thirty-three (91.7%) of 36 patients were treated in different hospitals, which limited our ability to obtain treatment methodology and results. We did not investigate whether the outbreak had started prior to 1 July 2008, because the physician did not report any patients with adverse effects before then. We could not compare patients with controls due to lack of consent and participation in this present study.

In conclusion, we report an outbreak of *M. abscessus* infection following TPI and/or EB that involved 36 patients. There needs to be more attention for invasive procedures management, including TPI and EB in rural clinics. Epidemiologic studies also are needed to explain the exact source of infection to prevent future outbreaks of *M. abscessus* infection.

## Figures and Tables

**Table 1:**
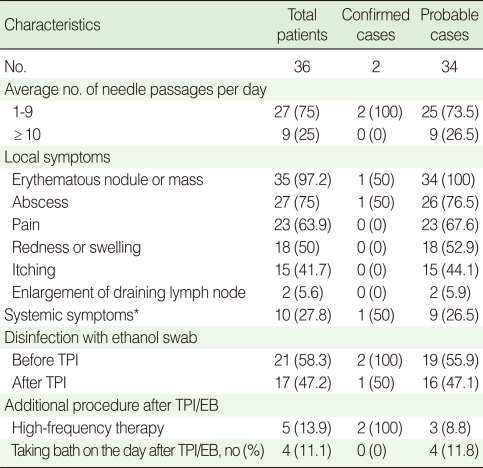
Clinical characteristics of patients with *Mycobacterium abscessus* infections following trigger point injection and/or epidural block

Values are presented as number (%). TPI, trigger point injection; EB, epidural block. ^*^Systemic symptoms include febrile sense, chill, and fatigue.
